# Explainable machine learning for predicting recurrence-free survival in endometrial carcinosarcoma patients

**DOI:** 10.3389/frai.2024.1388188

**Published:** 2024-12-06

**Authors:** Samantha Bove, Francesca Arezzo, Gennaro Cormio, Erica Silvestris, Alessia Cafforio, Maria Colomba Comes, Annarita Fanizzi, Giuseppe Accogli, Gerardo Cazzato, Giorgio De Nunzio, Brigida Maiorano, Emanuele Naglieri, Andrea Lupo, Elsa Vitale, Vera Loizzi, Raffaella Massafra

**Affiliations:** ^1^Laboratorio di Biostatistica e Bioinformatica, Fisica Sanitaria, I.R.C.C.S. Istituto Tumori “Giovanni Paolo II”, Bari, Italy; ^2^Ginecologia Oncologica, I.R.C.C.S. Istituto Tumori “Giovanni Paolo II”, Bari, Italy; ^3^Dipartimento di Medicina di Precisione e Rigenerativa e Area Jonica (DiMePRe-J), Università degli Studi di Bari “Aldo Moro”, Bari, Italy; ^4^Dipartimento Interdisciplinare di Medicina (DIM), Università degli Studi di Bari “Aldo Moro”, Bari, Italy; ^5^Dipartimento dell’Emergenza e dei Trapianti di Organi, Università degli Studi di Bari “Aldo Moro”, Bari, Italy; ^6^Laboratory of Biomedical Physics and Environment, Department of Mathematics and Physics "E. De Giorgi", Università del Salento, Lecce, Italy; ^7^Advanced Data Analysis in Medicine (ADAM), Laboratory of Interdisciplinary Research Applied to Medicine (DReAM), Università del Salento, Lecce, Italy; ^8^Oncologica Medica, Casa Sollievo della Sofferenza, San Giovanni Rotondo, Italy; ^9^Direzione Scientifica, I.R.C.C.S. Istituto Tumori “Giovanni Paolo II”, Bari, Italy; ^10^Dipartimento di Biomedicina Traslazionale e Neuroscienze (DiBraiN), Università degli Studi di Bari "Aldo Moro", Bari, Italy

**Keywords:** endometrial carcinosarcoma, recurrence-free survival, machine learning, explainable artificial intelligence, personalized medicine

## Abstract

**Objectives:**

Endometrial carcinosarcoma is a rare, aggressive high-grade endometrial cancer, accounting for about 5% of all uterine cancers and 15% of deaths from uterine cancers. The treatment can be complex, and the prognosis is poor. Its increasing incidence underscores the urgent requirement for personalized approaches in managing such challenging diseases.

**Method:**

In this work, we designed an explainable machine learning approach to predict recurrence-free survival in patients affected by endometrial carcinosarcoma. For this purpose, we exploited the predictive power of clinical and histopathological data, as well as chemotherapy and surgical information collected for a cohort of 80 patients monitored over time. Among these patients, 32.5% have experienced the appearance of a recurrence.

**Results:**

The designed model was able to well describe the observed sequence of events, providing a reliable ranking of the survival times based on the individual risk scores, and achieving a C-index equals to 70.00% (95% CI, 59.38–84.74).

**Conclusion:**

Accordingly, machine learning methods could support clinicians in discriminating between endometrial carcinosarcoma patients at low-risk or high-risk of recurrence, in a non-invasive and inexpensive way. To the best of our knowledge, this is the first study proposing a preliminary approach addressing this task.

## Introduction

Endometrial carcinosarcoma is a biphasic tumor with both carcinomatous (epithelial) and sarcomatous (mesenchymal) elements (Raffone et al., 2022). It is a rare, aggressive high-grade endometrial cancer, accounting for about 5% of all uterine cancers and nearly 20% of non-endometrioid endometrial carcinomas (Siegel et al., 2022). Although non-endometrioid tumors make up 10–20% of endometrial malignancies, they are responsible for over 40% of endometrial cancer deaths (Lu and Broaddus, 2020).

The treatment can be complex, including the need to perform surgery, platinum-based chemotherapy, and radiotherapy. Despite this, the prognosis remains poor (Travaglino et al., 2022). Median overall survival is less than 2 years, and the 5-year overall survival is under 30% (approximately 50% for early stage and 20% for advanced stage disease). Even patients with early-stage disease have a 45% 5-year recurrence rate and 50% 5-year disease-specific mortality (Toboni et al., 2021). The rising incidence and poor outcomes of endometrial carcinosarcoma highlight an unmet need of personalising the management of these challenging patients, in order to allow more informed and targeted decision-making even in the presence of a complex prognosis (Grasso et al., 2017).

Over the past few years, due to the increased availability of data and the greatest computing power, Artificial Intelligence (AI) has emerged as a potential tool to deal with this big data, with the aim of optimizing cancer research, improving clinical practice, and promoting precision in healthcare (Farina et al., 2022). Specifically, Machine Learning (ML) is a subfield of AI which exploits mathematical and statistical approaches to develop learning models able to detect hidden patterns in the data and to improve model performance (Cuocolo et al., 2020). Anyway, ML models are often related to the concept of “black box” whereby even the operators who engineered the model cannot explain the reasoning behind their predictions. Hence, eXplainable Artificial Intelligence (XAI) algorithms have been designed to enable users to understand and appropriately trust ML model decisions (Gunning et al., 2019; Doshi-Velez and Kim, 2017).

Recently, ML methods have been also employed in the identification of prognostic factors capable of predicting cancer survival and the risk of disease recurrence, with the purpose of supporting clinicians in optimizing the clinical follow-up plan (Wang et al., 2019). The ability of ML methods to handle survival data by modelling the relationship between the event of interest and the predictor variables, could allow the design of personalized therapeutic options, showing better accuracy than conventional statistical approaches (Mazzaki et al., 2021). As a matter of fact, ML algorithms are able to capture and shape non-linearities between variables without having to specify the form of the relationship between them *a priori*.

So far, in the state-of-the-art, several ML models able to predict early diagnosis, as well as response to therapy and disease recurrence in the gynaecological cancers field have been proposed (Fiste et al., 2022; Sheehy et al., 2023; Akazawa and Hashimoto, 2021). However, there is a lack of ML models to predict recurrence-free survival in patients affected by endometrial carcinosarcoma. To the best of our knowledge, in this study, we proposed the first explainable ML approach which addresses this task exploiting the predictive power of clinical and histopathological data, as well as chemotherapy and surgical information of 80 endometrial carcinosarcoma patients.

## Materials and methods

### Experimental dataset

From 1988 to 2021, a total of 80 female patients affected by endometrial carcinosarcoma were enrolled and monitored over time, with the purpose of supervising their clinical pathway and the prospective occurrence of a recurrence event. While monitoring, 26 patients (32.5%) have experienced the appearance of a recurrence, and 54 patients (67.5%) have not recurred.

For each patient, clinical and histopathological data, as well as chemotherapy and surgical information, were collected from the patients’ medical records. A total of 11 features were compiled, comprising the occurrence of a recurrent event (abbr. Recurrence, values: yes, no), age at diagnosis, tumor stage (abbr. Stage, values: I-II, III-IV), tumor hystotype (abbr. Type, values: homologous MMMT, heterologous MMMT), tumor size (abbr. size, values: ≤4cm, >4 cm), type of surgery (abbr. surgery, values: laparoscopic-LPS, laparotomic-LPT), having performed the omentectomy (abbr. omentectomy, values: yes, no), having performed the lymphadenectomy (abbr. lymphadenectomy, values: yes, no), chemotherapy scheme received (abbr. CT scheme, values: CBDCA, no CBDCA). The observation time, intended as the time in months between the date of diagnosis and either the appearance of a recurrence for recurrent patients or the last follow-up for non-recurrent patients, was also recorded for each patient.

An overview about the sample clinical properties is provided by Table 1.

**Table 1:** Clinical features distribution over the study population.

Feature	Distribution
Overall	80; 100%
Recurrence	
Yes (abs; %)	26; 32.5%
No (abs; %)	54; 67.5%
Age at diagnosis	
Median; [q_1_, q_3_]	67; [60, 74]
Stage	
I-II (abs; %)	45; 56.8%
III-IV (abs; %)	35; 43.8%
Type	
Homologous MMMT (abs; %)	33; 41.2%
Heterologous MMMT (abs; %)	47; 58.8%
Size	
≤ 4 cm (abs; %)	29; 36.2%
> 4 cm (abs; %)	51; 63.8%
Surgery	
LPS (abs; %)	4; 5%
LPT (abs; %)	76; 95%
Omentectomy	
Yes (abs; %)	23; 28.8%
No (abs; %)	57; 71.2%
Myometrial invasion	
Yes (abs; %)	72; 90%
No (abs; %)	8; 10%
Lymphadenectomy	
Yes (abs; %)	42; 52.5%
No (abs; %)	38; 47.5%
CT scheme	
CBDCA (abs; %)	60; 75%
No CBDCA (abs; %)	20; 25%
Observation time (mm)	
Median; [q_1_, q_3_]	24; [7.5, 72.5]

### Study design

We performed a ML recurrence-free survival analysis to estimate the time it takes for recurrence events to occur depending on the combination of values assumed by features.

The two most important notions on which survival analysis is based are the survival and hazard functions (Kartsonaki, 2016). The *Survival function* is defined as the probability of an event of interest T occurring after a specified time t:


$$S\left(t\right)=\mathrm{Pr}\left(T>t\right)$$

On the other hand, the *Hazard function* represents the likelihood for an individual to experience the event in a short interval of time t+Δt, given that the event has not occurred before time t:


$$h\left(t\right)=\frac{\mathrm{Pr}\left(t<T\leq t+\Delta t|T>t\right)}{\Delta t}$$

A starting point for the analysis needs to be also defined, and the beginning of each subject’s observation time coincides with this starting point, time at which all subjects have the same risk equals to zero of the event occurring.

In this study, we implemented a stratified 5-fold cross-validation scheme over 5 rounds starting from clinical data of all patients enrolled, by dividing the population into strata, so that the right number of cases are sampled from each stratum to guarantee that the test set is representative of the entire population.

The first step consisted in adopting a feature selection approach based on a recursive feature elimination technique to identify a subset of relevant features for the outcome prediction. Starting from the original set of features, this technique allows to identify the optimal subset by recursively eliminating less important features by means of a linear regression. Features are consequently ranked according to their estimated significance, and only the most important ones are employed for further steps (Ambusaidi et al., 2016).

Then, we trained a Gradient Boosting (GB) algorithm to determine how the hazard function varied according to the associated features previously selected. The GB algorithm is a non-parametric supervised learning belonging to the category of ensemble methods: it sequentially combines the predictions of multiple simple models named *base learners*, allowing each new learner to correct the previous one and, consequently, to reinforce the overall model. This process enables the minimization of a specific loss function using a well-defined base learner (Nguyen, 2019). In this work, we optimized the partial likelihood loss of the Cox’s proportional hazards model by means of a regression tree base learner (Kleinbaum and Klein, 2012; Breiman et al., 2017). Specifically, a total of 100 estimators were employed. The model was trained setting the other hyperparameters as default (Pölsterl, 2020).

The discrimination power of the above-mentioned model was then evaluated in terms of the Concordance-index (C-index), that represents the model’s ability to correctly provide a reliable ranking of the survival times based on the individual risk scores (Longato et al., 2020).

According to GB model predictions, for each patient in the test set both a predicted survival and a predicted cumulative hazard function were estimated and depicted. These are two stepwise functions in which the occurrence of one or more events is represented by a vertical drop or slope, respectively.

Finally, we adopted a XAI algorithm named Surv Local Interpretable Model-agnostic Explanations (SurvLIME), to explain the contribution of each of the most significant features to the decision of the ML survival model, both at patient and at all dataset levels. In both cases, this method allows to compute local interpretability by providing a ranking among the set of features, even considering the time space to give explanations with the goal of detecting possible dependencies between the features and the time (Kovalev et al., 2020). Particularly, to make these final predictions and explanations, we identified the most performing model among all models trained within the 5-fold cross-validation scheme, and we considered the same test set preliminarily identified according to the cross-validation framework. Therefore, only a subset was considered in this process to avoid overly optimistic estimates.

The idea behind the SurvLIME algorithm is to approximate the output produced by the ML survival model which has to be explained, by the output produced by a model belonging to a set of explanation models. Specifically, this approximation model is trained on new perturbed samples generated with the corresponding predictions of the ML survival model, by solving an optimization problem which minimizes the distance between the explanation and the prediction of the ML survival model. The approximation model adopted by the SurvLIME algorithm is the Cox proportional Hazards model, that is a semi-parametric survival algorithm whose output is the result of a multiple regression (Kleinbaum and Klein, 2012).

All the analysis steps have been performed by using Python.

## Results

**Figure 1:**
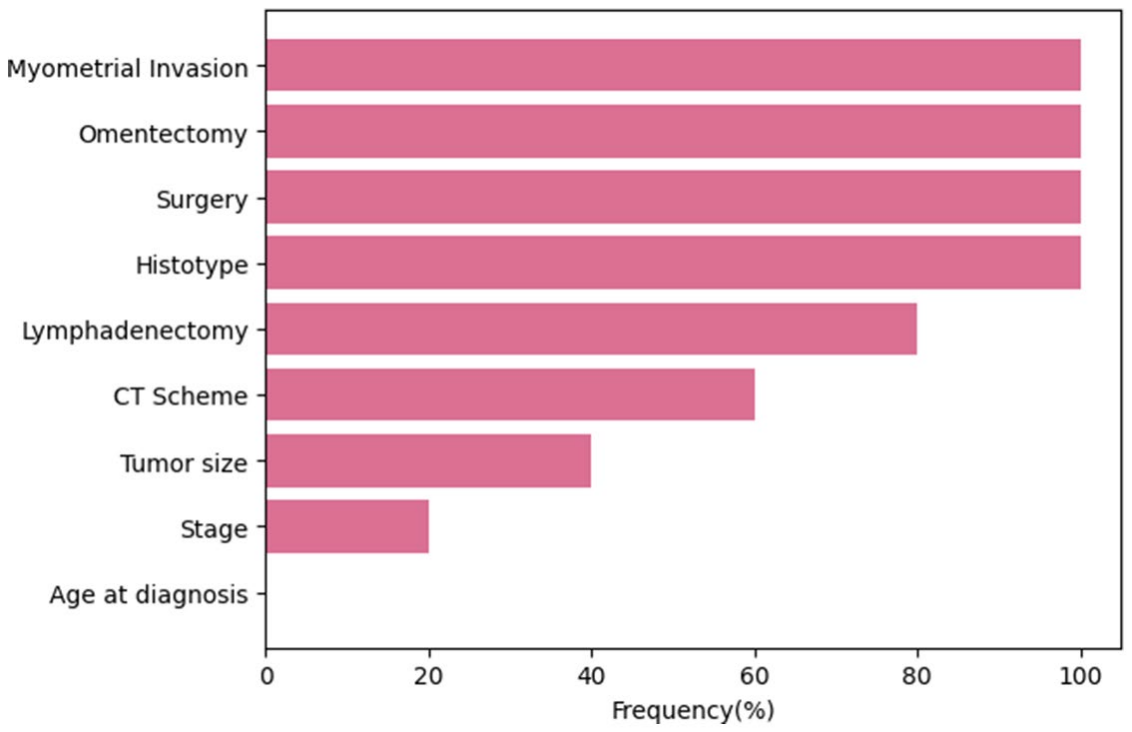
Frequency of selection of each feature over all rounds.

At the end of each cross-validation round, features were ranked in descending order according to their estimated significance, and only features with a rank ≤3 were selected. The frequency of selection of each feature within all rounds of the cross-validation procedure is shown in Figure 1. Four variables, namely, myometrial invasion, omentectomy, surgery and histotype were always selected, presenting a frequency equals to 100%. Conversely, the age at diagnosis has never been selected as an important feature over the training process.

The designed ML survival model, trained on features resulted important by turns, was able to well describe the observed sequence of events, and its discrimination power was evaluated in terms of C-index along with its 95% confidence interval: 70.00% [59.38–84.74].

**Figure 2:**
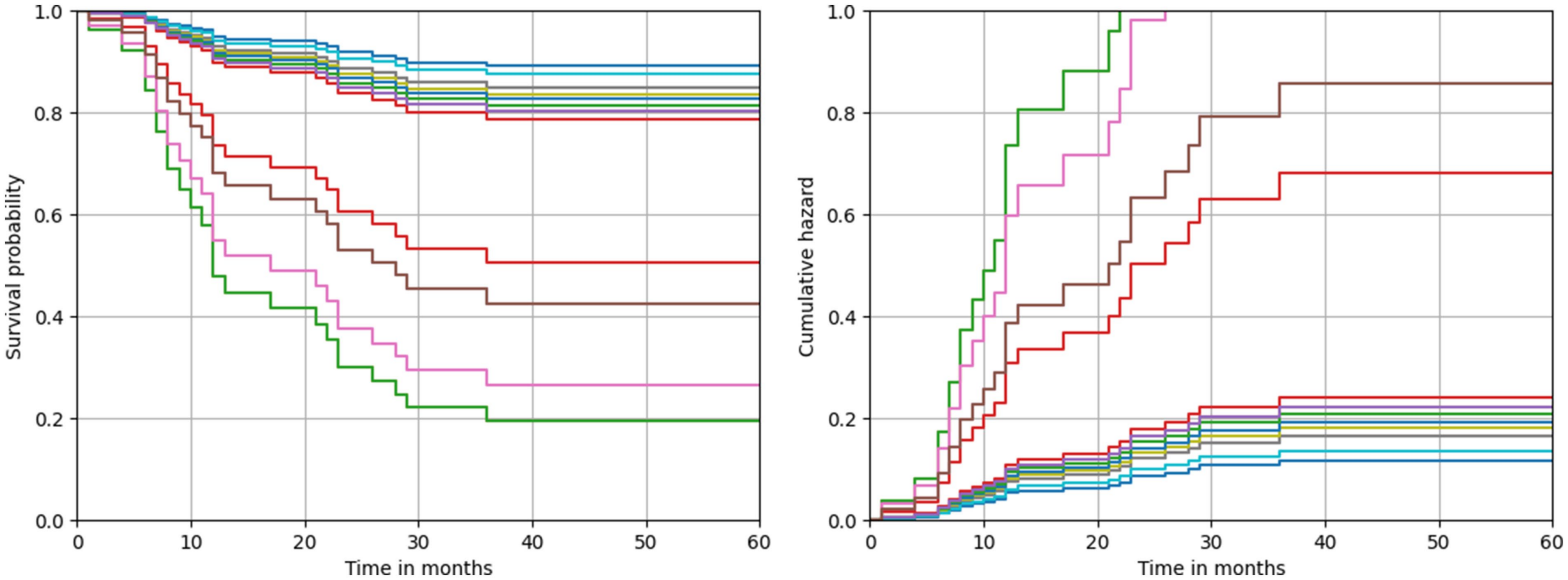
Predicted survival and cumulative hazard functions estimated by the best performing model.

The model predictions also allowed to describe both a survival and a cumulative hazard function for each patient. The respective functions estimated by means of the best performing model are depicted in Figure 2. Due to the specularity of these functions, in both cases the curves resulted well separated into two groups. The first group of patients was characterized by a lower survival probability and, consequently, a higher risk of recurrence since the first months after diagnosis. Conversely, patients belonging to the second group were identified by a survival probability always greater than 80%, and which trend remained constant even after several months after diagnosis. A comparison highlighted that patients with a higher risk of recurrence all share the following feature values: a heterologous MMMT type, a CBDCA CT scheme and a laparotomic-LPT surgery performed.

**Figure 3:**
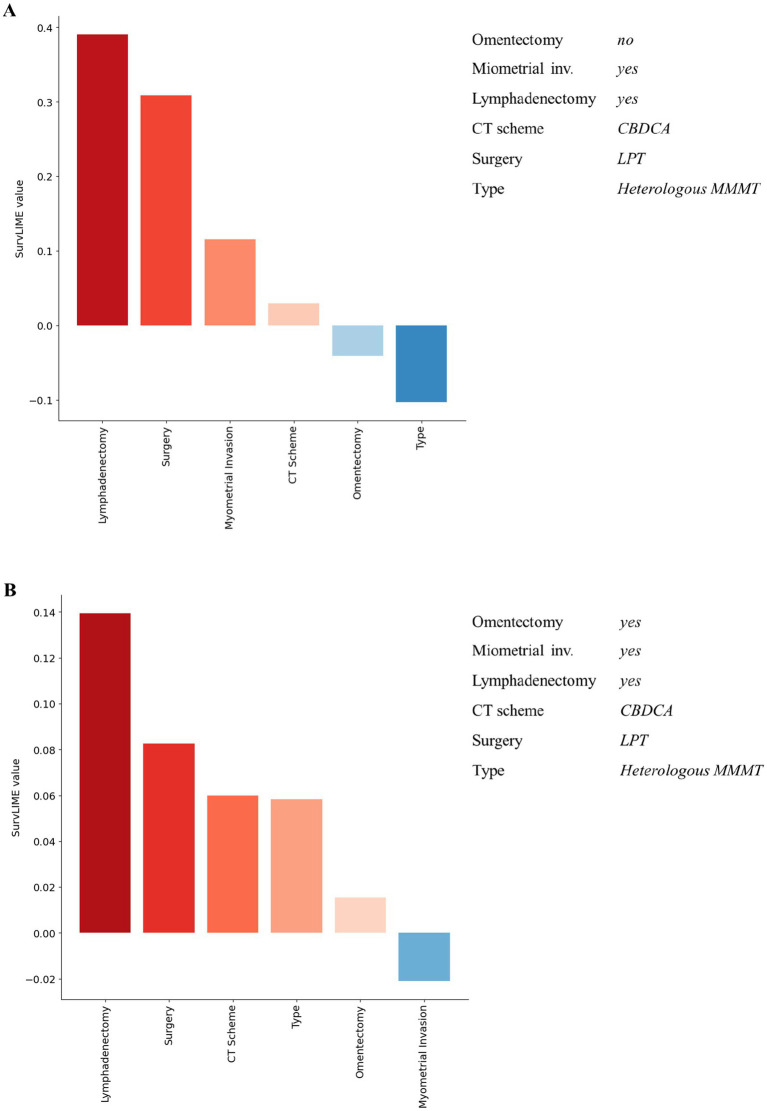
Examples of explanation at patient level for recurrent patients associated with a high predicted hazard. Red colour and blue colour palette indicate positive and negative contributions for increasing the Cumulative Hazard Function, respectively.

**Figure 4:**
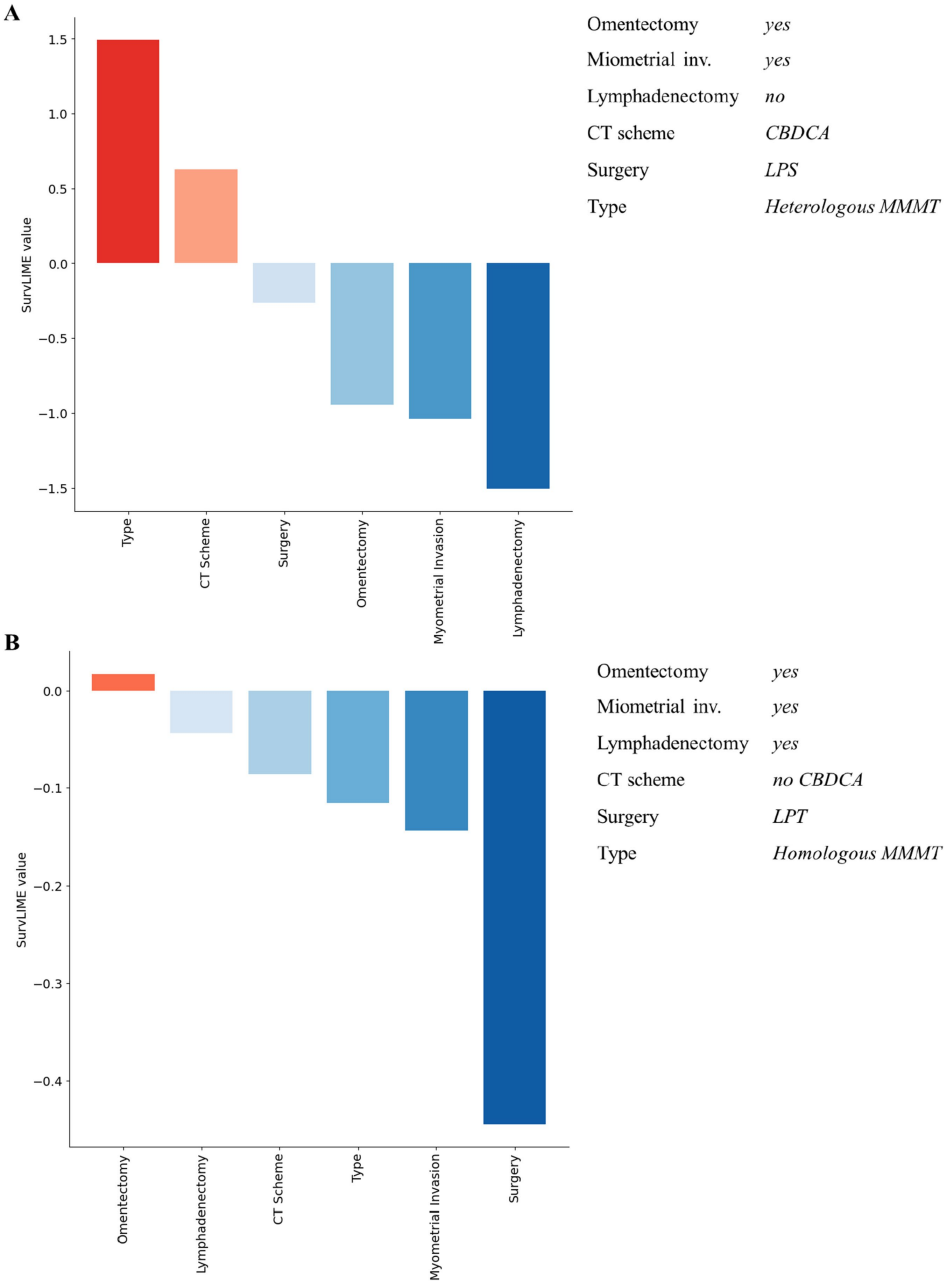
Examples of explanation at patient level for non-recurrent patients associated with a low predicted hazard. Red colour and blue colour palette indicate positive and negative contributions in increasing the Cumulative Hazard Function, respectively.

Despite the good performances of our ML survival model in predicting recurrence-free survival, the reasoning behind its predictions is unknown. To this aim, we provided local explanations of the contribution of important features to the model prediction, both at patient and all dataset level. Figures 3, 4 shows some examples of explanations at patient level. Each explanation consists of a feature importance diagram in which the feature contributions to the outcome are displayed in descending order, using a red colour palette for the features that increase the Cumulative Hazard Function and a blue palette for those that decrease it.

Considering Figure 3, for both patients the ML survival model correctly returned a high predicted hazard. This can be related to having performed the lymphadenectomy, a CBDCA CT scheme and a laparotomic-LPT surgery. On the other hand, patients illustrated in Figure 4 are related to a correctly low predicted hazard. In these cases, explanation diagrams highlighted how differences among their feature values are related to different feature contributions in terms of weight to the outcome prediction.

**Figure 5:**
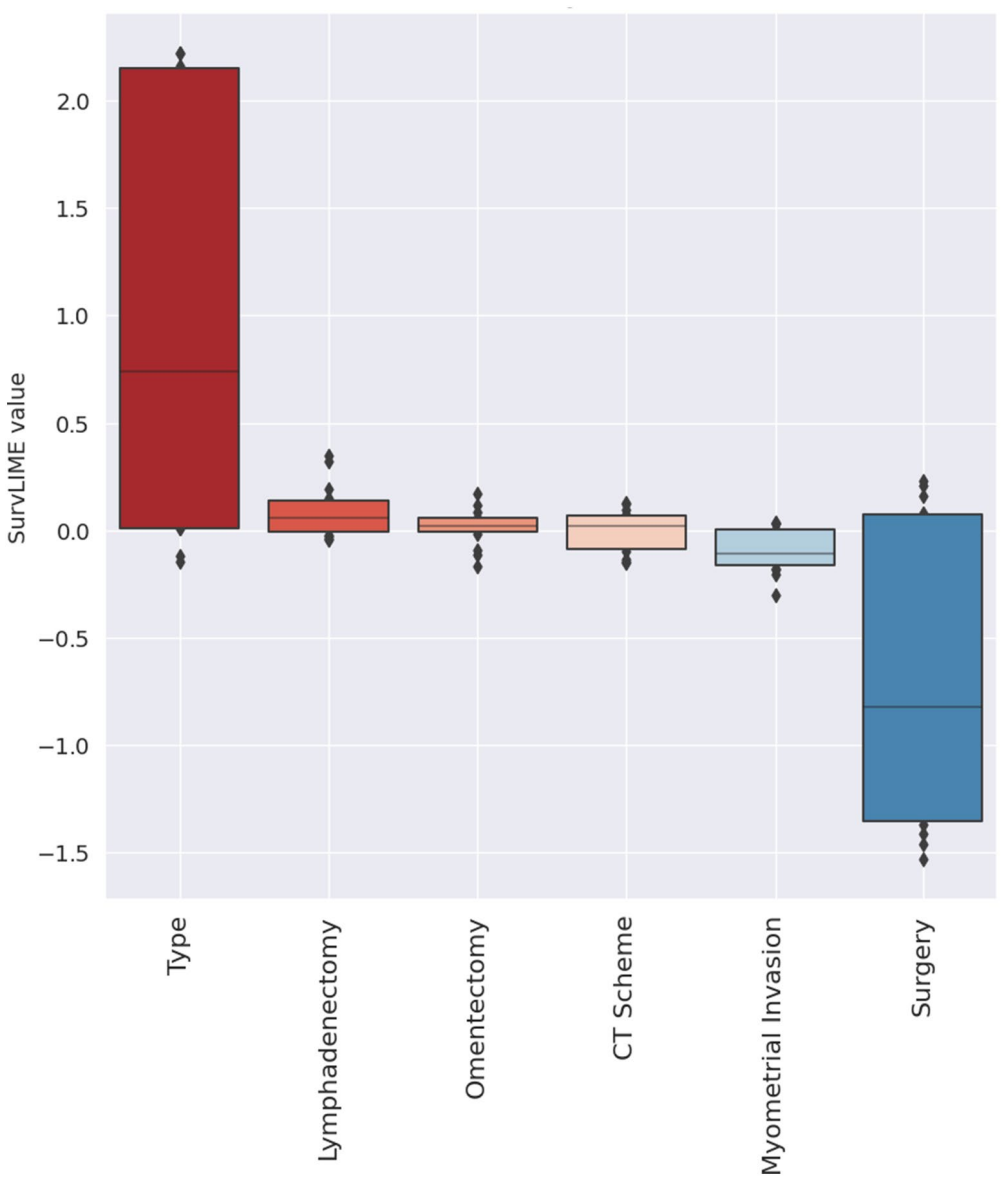
Explanation at all dataset level. Red colour and blue colour palette indicate positive and negative contributions in increasing the Cumulative Hazard Function, respectively.

Lastly, Figure 5 depicts the local explanation at all dataset level. In this feature importance diagram, feature contributions are displayed in descending order by means of box plots representing the feature contribution distributions computed over the entire dataset. The feature positive or negative contribution in increasing the Cumulative Hazard Function is pictured by a red colour or blue colour palette, respectively.

## Discussion

Uterine carcinosarcoma is a high-grade tumor including both epithelial and mesenchymal malignant cell components. Typically, the former shows low differentiation and a mix of characteristics, possibly displaying traits like endometrioid, clear-cell, or serous features. Tumor cells may organize in gland-like structures. The latter can resemble either endometrial stromal sarcoma or leiomyosarcoma, known as “homologous,” or it may exhibit features akin to specialized connective tissues outside the uterus, such as muscle, cartilage, and bone, termed “heterologous.” In both scenarios, angiolymphatic invasion is frequently observed (Bogani et al., 2023).

Despite surgical treatment and timely adjuvant multimodal therapy, more than half of the cases of endometrial carcinosarcoma will recur within the first 2 years (Concin et al., 2021). The management of the recurrent disease is highly personalized and should consider several factors, such as the performance status of the patient, the size and sites of recurrences, and prior therapies (Pezzicoli et al., 2021). Importantly, it depends on whether the relapse is locoregional, oligometastatic, or disseminated and, second, on whether the patient has already received radiotherapy, as radiotherapy rechallenge is generally avoided for safety reasons. Again, the best treatment approach is multimodal. Patients with recurrent disease (including peritoneal and lymph node relapse) should be considered for surgery only if it is anticipated that complete removal of macroscopic disease can be achieved with acceptable morbidity and be treated in specialized centres (Beckmann et al., 2021). External beam radiotherapy can be used in radiotherapy-naïve patients or those who had received only prior vaginal brachytherapy. Immunotherapy (with or without tyrosine kinase inhibitor) is the emerging preferred second-line systemic treatment. After the failure of immunotherapy, chemotherapy alone (generally mono-chemotherapy) is the preferred treatment in cases of disseminated metastases (Abu-Rustum et al., 2021).

Owing to the rare and aggressive nature of endometrial carcinosarcoma, the complexity of its management both at diagnosis and recurrence, as well as its high recurrence rate (Amant et al., 2005), identifying an ensemble of prognostic factors able to accurately predict recurrence-free survival in patients affected by this malignancy could allow more informed and targeted decision-making, enabling proactive clinical management even in the presence of a complex prognosis. Actually, the ability to identify patients at a major risk of recurrence at an early stage could allow clinicians to tailor treatment plans, both adopting more aggressive strategies such as intensive or combination chemotherapy protocols, adjuvant radiotherapy or experimental approaches in patients with a high probability of recurrence and sparing low-risk patients from unnecessary interventions. Moreover, an accurate predictive model can guide the frequency and intensity of clinical follow-up, allowing an early detection of recurrence and improving the likelihood of disease control. Finally, thanks to predictive modelling, it is possible to more selectively enrol patients which may be candidates for clinical trials of new drugs or experimental therapies, especially when standard options have obvious limitations.

Over the past years, several efforts have been made to develop a greater awareness and deeper understanding of endometrial carcinosarcoma pathogenesis, with the purpose of identifying new targeted therapies and providing specific guidelines for the management of this tumor (Bogani et al., 2023). Besides, endometrial cancer treatment has provided new changes by incorporating biological, clinical, genomic, and clinico-pathologic characteristics of the women affected by this tumor, and recent studies showed that molecular targets such as L1CAM (L1 cell adhesion molecule) plays an important role as prognostic factor and could provide a potential useful tool for tailoring the need of adjuvant therapy (Giannini et al., 2024; Vizza et al., 2021). As well, a prognostic nomogram to predict the overall survival rate in endometrial carcinosarcoma patients by exploiting lymph-node metastasis information has been proposed (Gao et al., 2021).

However, there is a lack of research studies focused on the prediction of disease recurrence risk.

In this study, we proposed the first explainable ML method designed to predict recurrence-free survival in patients affected by endometrial carcinosarcoma. The nested feature importance approach allowed us to identify the most relevant variables for this outcome prediction. Accordingly, promising results were achieved in providing a reliable ranking of the survival times based on the individual risk scores (C-index: 70%). Finally, with the aim to enable clinicians to understand the reasoning behind the ML model predictions, the implemented XAI algorithm computed the contribution of each of the most significant features to the model decision, both at patient and at all dataset levels.

To conclude, the proposed explainable ML model represents the first effort in devising an artificial intelligence-based tool to be enclosed in clinical practice to support clinicians in discriminating between endometrial carcinosarcoma patients at low-risk or high-risk of recurrence in a non-invasive and inexpensive way, also providing an intelligible explanation on how the clinical characteristics considered for those patients contributed to the estimated risk. Accordingly, the ability of this model in detecting the risk for a patient of experiencing recurrence could aid clinicians to personalise therapeutic options, by candidating high-risk patients to adjuvant chemotherapy and saving low-risk patients from unnecessary aggressive treatments.

Besides, a limitation of our study deals with its retrospective design and the limited dimension of the dataset. As far as the limited dataset size, this could affect the robustness and generalizability of the ML model which generally require larger dataset, in contrast to what classic survival approaches, such as Cox regression, need. However, the advantages in exploiting ML techniques rather than classical methods are the increased flexibility, the ability to adapt to non-linear relationships, together with improved predictive performances. Actually, relationships between variables are often non-linear or complex, and some effects may depend on interactions between them. ML algorithms are able to capture and shape these non-linearities and interactions without having to specify the form of the relationship between variables *a priori*. Definitely, employing ML models with larger datasets, it could be possible to achieve higher performances and improve the model. For this purpose, in our future work we will collect an external dataset for prospective validation, in order to establish documented evidence that the model is able to consistently produce the desired results within predetermined specifications and quality attributes.

## Data Availability

The data analyzed in this study is subject to the following licenses/restrictions: data from this study are available upon request since data contain potentially sensitive information. The data request may be sent to the scientific direction (e-mail: dirscientifica@oncologico.bari.it).
